# Pharmacokinetics and Pharmacodynamics of Meropenem by Extended or Continuous Infusion in Low Body Weight Critically Ill Patients

**DOI:** 10.3390/antibiotics10060666

**Published:** 2021-06-03

**Authors:** Sonia Luque, Adela Benítez-Cano, Leire Larrañaga, Luisa Sorlí, María Eugenia Navarrete, Nuria Campillo, Jesús Carazo, Isabel Ramos, Ramón Adalia, Santiago Grau

**Affiliations:** 1 Department of Pharmacy, Hospital del Mar, IMIM (Hospital del Mar Research Institute), Universitat Autonoma de Barcelona, 08003 Barcelona, Spain; mnavarrete@psmar.cat (M.E.N.); NCampillo@parcdesalutmar.cat (N.C.); sgrau@parcdesalutmar.cat (S.G.); 2 Department of Anaesthesiology and Surgical Intensive Care, Hospital del Mar, IMIM (Hospital del Mar Research Institute), 08003 Barcelona, Spain; llarranaga@psmar.cat (L.L.); jcarazo@psmar.cat (J.C.); mramos@psmar.cat (I.R.); radalia@psmar.cat (R.A.); 3 Department of Infectious Diseases, Hospital del Mar, IMIM (Hospital del Mar Research Institute), Universitat Autonoma de Barcelona, 08003 Barcelona, Spain; lsorli@psmar.cat

**Keywords:** antibiotics, pharmacokinetics, pharmacodynamics, low body weight, meropenem

## Abstract

*Background*: Pathophysiological changes such as extreme body weights in critically ill patients with severe infections may alter the pharmacokinetics (PK) of antimicrobials, leading to treatment failure or toxicity. There are almost no PK data on meropenem in critically ill patients with low body weight (LwBW) and therefore information is lacking on the most appropriate dosing regimens, especially when administered by extended infusion. *Objectives*: To assess if the current administered doses of meropenem could lead to supratherapeutic concentrations in LwBW patients and to identify the factors independently associated with overexposure. Methods: A matched case-control 1:1 study of surgical critically ill patients treated with meropenem administered by extended or continuous infusion and undergoing therapeutic drug monitoring was conducted. Cases (patients with LwBW (body mass index (BMI) < 18.5 kg/m^2^)) were matched with normal body weight controls (NBW) (patients with BMI ≥ 18.5 kg/m^2^ and ≤30 kg/m^2^)) by age, gender, baseline renal function and severity status (APACHE II score). A 100% fT > MIC was considered an optimal pharmacokinetic/pharmacodynamic (PK/PD) target and 100% fT > 10 × MIC as supratherapeutic exposure. *Results*: Thirty-six patients (18 cases and 18 controls) were included (median (range) age, 57.5 (26–75) years; 20 (55.6% male)). Meropenem was administered by 6 h (extended) or 8 h (continuous) infusion at a median (range) daily dose of 5 (1–6) g/day. Similar median meropenem trough plasma concentrations (Cmin,ss), measured pre-dose on day three to four of treatment) were observed in the two groups (19.9 (22.2) mg/L vs 22.4 (25.8) mg/L, *p* > 0.999). No differences in the proportion of patients with an optimal or a supratherapeutic PKPD target between cases and controls were observed. A baseline estimated glomerular filtration rate (eGFR) < 90 mL/min was the only factor independently associated with a supratherapeutic PK/PD target. *Conclusions*: LwBW seems not to be a risk factor for achieving a supratherapeutic PK/PD target in critically ill patients receiving meropenem at standard doses by extended or continuous infusion.

## 1. Introduction

Meropenem is a carbapenem with a broad spectrum of activity against a great variety of Gram-positive and Gram-negative pathogens. Its good penetration into fluids and tissues makes meropenem a common choice for the treatment of severe infections in critically ill patients [1,2]. As a β-lactam, meropenem exhibits time-dependent pharmacodynamics (PD), being the fraction of the dosing interval that free plasma concentrations are above the minimum inhibitory concentration (MIC) (fT > MIC), the pharmacokinetic/pharmacodynamics (PK/PD) index related to its antimicrobial efficacy [3]. Although the maximal bactericidal activity for meropenem was initially associated with a fT > MIC of 40–50% in in vitro studies [4], a higher PK/PD target of 100% fT > MIC has been suggested for critically ill patients in clinical studies to ensure bactericidal activity and minimize the emergence of antimicrobial resistance [5,6,7,8]. Administration of meropenem by extended (EI) or continuous infusion (CI), rather than intermittent infusion, may be helpful for increasing fT > MIC. The use of higher doses and prolonged infusions are strategies especially recommended in patients infected with highly resistant bacterial strains in order to improve clinical outcomes and reduce the risk of recurrence [7,8,9].

Numerous studies have evaluated the pharmacokinetics (PK) of meropenem in critically ill patients with different physiopathological alterations (sepsis, renal dysfunction, renal replacement therapies, obesity) [10,11]. Extreme body weights can also be associated with physiological changes affecting the PK properties of antimicrobials. Critically ill patients can have excessively high or low body weight (LwBW), and while several studies have described the effects of obesity on the PK behavior of different antimicrobials [10,12,13], information in patients with LwBW is very scarce and mostly drawn from pediatric patients [14,15,16]. LwBW states occur more often in the elderly, at end-life-states and in patients with cancer, dementia and malabsorption syndromes [17]. This population may show a reduction in the volume of distribution of hydrophilic drugs, such meropenem, that could lead to high and potentially toxic plasma concentrations. In fact, dosing recommendations for this population are lacking and it is unknown whether the administration of standard doses may be excessive, especially when beta-lactams are administered by prolonged infusions.

The aim of this study was to analyze the PK of meropenem administered by EI/CI in a cohort of LwBW critically ill patients and to assess whether the current administered doses may lead to supratherapeutic concentrations. For this purpose, we compared the PK of meropenem in a cohort of critically ill patients with LwBW (cases) with a control group of normal body weight (NBW) patients. As a secondary objective, we also analyzed the predictive risk factors for achieving a supratherapeutic PK/PD target.

## 2. Material and Methods

### 2.1. Setting and Study Population

This single-center, retrospective, observational PK study was conducted in a tertiary surgical intensive care unit (ICU) at Parc de Salut Mar, Barcelona, between January 2017 and October 2020. We included patients with LwBW (body mass index (BMI) < 18.5 kg/m^2^) [18] treated with meropenem administered by EI/CI (by 6 h or 8 h) and undergoing therapeutic drug monitoring (TDM) of meropenem. Controls were matched with cases by age (±10 years), sex, renal function (±20% baseline estimated glomerular filtration rate (eGFR) [19]) and severity status (±5 points in Acute Physiology and Chronic Health Evaluation (APACHE II) score [20]).

Ethics approval was obtained from our local Ethics Committee (Comitè Etic d’Investigació, CEIC-Parc de Salut Mar, approval number 2020/9601). The need for written consent was waived due to the observational retrospective nature of the study.

Exclusion criteria were previous use of carbapenems within 15 days to avoid potential residual meropenem plasma concentrations, patients receiving meropenem by intermittent infusion, those on renal replacement therapy and patients with severely impaired liver function (cirrhosis grade C by Child-Pugh classification) [21].

Demographic, clinical and laboratory data were collected in all included patients. 

Clinical cure was defined as survival and resolution of all signs and symptoms related to the infection during follow up and a decreased in C-reactive protein or procalcitonin levels > 80% from baseline (7 to 10 days after treatment initiation). Microbiological eradication was defined as eradication of the microorganism(s) cultured from biological samples at baseline and at the end of treatment.

Adverse events potentially associated with meropenem were collected, such as gastrointestinal (diarrhea, nausea/vomiting, constipation), neurological (headache, insomnia, agitation, delirium, confusion, dizziness, seizure, nervousness, paresthesia, hallucinations, somnolence), drug-induced liver injury (DILI) (increased alanine transaminase, aspartate transaminase, alkaline phosphatase, lactate dehydrogenase and bilirubin) or Clostridioides difficile-associated diarrhea.

### 2.2. Study Protocol

#### 2.2.1. Dosing Regimen and PK Sampling

The meropenem dose (Meropenem Accordpharma^®^; Accord Healthcare, S.L.U. Barcelona, Spain) was selected by the treating physician and was administered by EI (6 h infusion) or CI (8 h infusion). In most patients, an initial loading dose of 2 g was administered, and dosage adjustments were made based on renal function [22] (eGFR > 50 mL/min/m^2^ = no changes; eGFR 25-50 mL/min/m^2^ = 1g every 8 h; eGFR < 25 mL/min/m^2^ = 0.5g every 6 h; eGFR < 10 mL/min/m^2^ = 0.5g every 12 h), when indicated. After 3–4 days of meropenem treatment, blood samples were obtained at pre-dose (Cmin,ss) for TDM.

#### 2.2.2. Pharmacokinetic/Pharmacodynamics (PK/PD) Analysis

Meropenem concentrations were measured using a validated high-performance liquid chromatography method [23] at the Pharmacy Department of Hospital del Mar. The assay was linear from 0.5 to 80 mg/L. Imprecision and inaccuracy were ≤15% at high, medium and low concentrations. The limit of quantification was 0.5 mg/L.

An optimal PK/PD target was defined as (100% fT ≥ 1–10 × MIC) while a 100 %fT > 10 × MIC was considered as supratherapeutic exposure [24]. Threshold for nephro and neurotoxicity (Cmin,ss > 44.5 mg/L and > 64.2 mg/L, respectively) were also considered based on the meropenem concentrations described in previous studies [25].

The PK/PD target was calculated considering the real MIC of the isolated pathogen or by using the European Committee on Antimicrobial Susceptibility Testing (EUCAST) susceptibility cut-off point of 2 mg/L, in those patients without an available MIC value [26].

Descriptive data are expressed as absolute and relative frequencies for categorical variables and medians (interquartile range, IQR) for quantitative variables. Continuous variables were compared using the Student *t* test or Mann–Whitney *U* test as appropriate and dichotomous variables were compared using the *X*^2^ test or Fisher exact test. Univariate analysis was performed to determine the association between each potential variable and the achievement of a supratherapeutic PK/PD target of meropenem. Those variables that were found to be statistically significant or with a *p* value < 0.15 in the univariate analysis were entered into a multivariate logistic regression model to calculate the adjusted odd ratio. A *p*-value < 0.05 was considered statistically significant. The SPSS version 22.0 statistical package (IBM, Armonk, NY, USA) was used throughout.

## 3. Results

During the study period, 27 patients with LwBW were included in the meropenem TDM program. Eighteen case patients fulfilled the inclusion criteria and were included in the study; 10 (55.6%) were male, and the median age was 61.5 (26–75) years. Six patients were excluded due to the need of renal replacement therapy, one patient presented acute on chronic liver failure and in two patients blood samples could not be obtained and meropenem concentrations were not available. Eighteen patients with NBW were matched as control group. In the whole group, most patients (22 (61.1%)) had sepsis or septic shock diagnosis at the beginning of meropenem treatment. The focus of infection in the LwBW group was: Respiratory in 12 (66.7%), abdominal in four (22.2%), urinary tract in one (5.6%) and neurological in one (5.6%) patients. In the NBW group, the focus of infection was respiratory in five (27.8%), abdominal in five (27.8%), urinary tract in three (16.7%), skin and soft tissues in three (8.3%) and neurological in two (11.1%) patients. In the LwBW group, eleven patients (61.1%) had a confirmed Gram-negative bacteria infection: Three extended-spectrum beta-lactamase (ESBL) *Escherichia coli* (MIC < 0.25 mg/L for meropenem), three *Klebsiella oxytoca* (two of them ESBL (MIC < 0.12 mg/L)), six *Pseudomonas aeruginosa* (three susceptible (MIC < 1 mg/L) and three extensively drug resistant (MIC 16–32 mg/L)) and one *Proteus mirabilis* (MIC < 2 mg/L). In the NBW group, eleven (61.1%) patients had a confirmed Gram-negative bacteria infection: Six *E. Coli* (three of them ESBL, MIC < 0.25 mg/L), three ESBL *K. pneumoniae* (MIC < 0.1 mg/L), two *P. aeruginosa* (CMI < 1 mg/L), four Enterobacter (3 *Enterobacter cloacae* (MIC < 0.12 mg/L) and one *Morganella morganii* (MIC < 0.016 mg/L). Two patients in the LwBW group and four patients in the NBW group had a polymicrobial infection.

Meropenem was administered by 6/8 h EI/CI three times daily in all patients (by 6 h in 23 (63.9%) patients and by 8 h CI in 13 (36.1%). The doses of meropenem given were 6 g/day in 18 patients (50%), between 3 to 4 g/day in 13 patients (36.1%) and ≤2 g/day in five patients (13.9%).

Table 1 shows the demographic and clinical variables of the included patients.

Clinical cure was achieved in 16 (88.9%) patients in the LwBW group and in 15 (83.3%) patients in the NBW group. 

**Figure 1:**
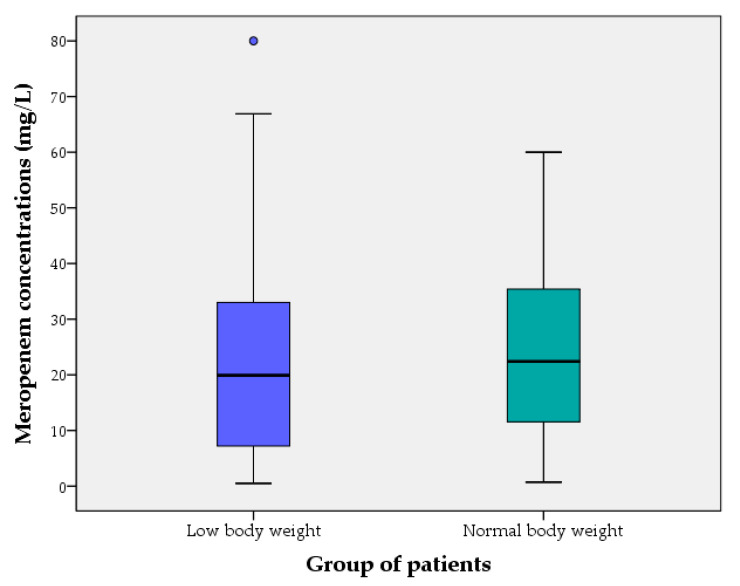
Meropenem plasma concentrations at steady state (mg/L) of cases (LwBW patients) and controls (NBW patients). The boxes represent the 25th, 50th and 75th percentiles.

**Figure 2:**
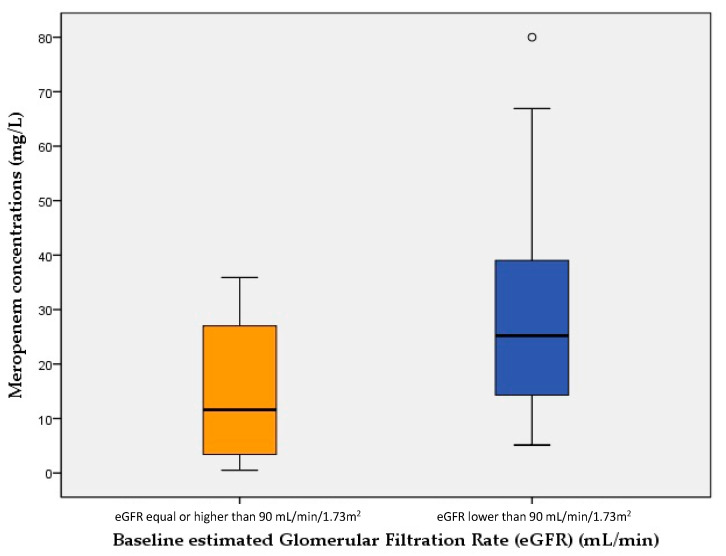
Meropenem plasma concentrations at steady state (mg/L) of patients with a baseline estimated Glomerular Filtration Rate (eGFR) < 90 mL/min and those with an eGFR ≥ 90 mL/min. The boxes represent the 25th, 50th and 75th percentiles.

Figure 1 and Figure 2 show meropenem plasma concentrations at steady state (mg/L) of cases (LwBW patients) and controls (NBW patients) comparing different baseline eGFR.

Table 2 shows meropenem concentrations, PK/PD targets and toxicity data comparing cases and controls.

Meropenem concentrations of the two patients who experienced DILI were 15.4 and 80 mg/L, respectively.

Eight of the twenty-five patients (32%) with a supratherapeutic PK/PD target had acute renal failure (ARF) at the start of meropenem therapy but they were also receiving an adjusted lower dose compared with those with preserved renal function (3 g/day (1–5) vs 6 g/day (3–6)). The two patients not achieving a 100%fT ≥ 1–10 MIC presented augmented renal clearance (eGFR 148 and 130 mL/min/1.73 m^2^, respectively).

In the univariate analysis, patients achieving a supratherapeutic PK/PD target had a lower baseline eGFR, a higher frequency of ARF, a lower MIC values and they presented more frequently septic shock. However, the presence of a LwBW was not statistically significant. In the multivariate analysis, only the presence of a baseline eGFR < 90 mL/min was identified as independent risk factors for achieving a supratherapeutic PKPD target (OR: 6.767 (95% CI 1.036–44.211), *p* = 0.046). Although a tendency to a lower MIC value for overexposure was also observed (OR: 0.310 (95% CI 0.094–1.018), *p* = 0.05), this result did not reach statistical significance. 

TDM recommendations were a dosage reduction (decrease dose by 25–50% with same dosing frequency) in six patients (33.3%) in the LwBW group and in four patients (22.2%) in the NBW group, dose increases (increase dose by 25–50% with same dosing frequency) in one patient (5.6%) in the LwBW group and in two patients (11.1%) in the NBW group and maintenance in seven patients (38.9%) in the LwBW group and in four patients (22.2%) in the NBW group. In the 13 remaining patients, the treatment had been stopped before a dosing recommendation could be made. 

## 4. Discussion

To our knowledge, this is the first study whose main objective is to assess the pharmacokinetics of meropenem administered by EI/CI in a cohort of underweight critically ill adult patients in comparison with normal weight patients. Several studies have described the effects of obesity on the PK behavior of different antimicrobials [10,12,13], confirming that obese patients have a higher volume of distribution than non-obese patients. However, standard doses seem to be sufficient to achieve optimal PK/PD targets and no doses adjustments are needed.

In patients with LwBW, the opposite condition, PK data or dosing recommendations are lacking. In this study, both cases (LwBW) and controls (NBW) were treated with meropenem standard doses and consequently patients in the LwBW group received a higher meropenem daily dose/actual body weight. However, no statistical differences in the median trough meropenem concentrations at steady state between the two groups were observed. In the same way, a similar proportion of patients achieved a therapeutic, supratherapeutic or infratherapeutic PK/PD target in both groups.

One important finding of this study is the fact that only 25% of patients in the whole cohort achieved an optimal PK/PD target (considering 100%fT ≥ 1–10 × MIC [29]), and in only two patients (5.6%, one of each group) the plasma exposure was suboptimal. On the contrary, more than 60% of patients showed what has been defined as overexposure (100%fT > 10 × MIC). However, it has to be considered that most of the bacterial isolates had low MIC values for meropenem, which lead to a high probability of achieving a supratherapeutic PK/PD target. In addition, although no differences in the frequency of patients with a supratherapeutic PK/PD target were found between cases and controls, it has to be considered that the individual MIC values of the bacterial isolates in the NBW group were generally lower.

Even though there was a high frequency of patients with a supratherapeutic exposure, only a few patients achieved meropenem concentrations that were above the proposed toxicity thresholds (three patients in the LwBW group and just one patient in the NBW group). Unfortunately, the limited sample size prevented us from drawing any conclusion about if LwBW patients may be at a higher risk for achieving potentially toxic meropenem plasma concentrations. From a PD point of view, concentrations of antimicrobials greater than four to five times MIC seem not to add any benefit in antibiotics with a time-dependent bacterial killing. In the study of Abdulla A. et al. [24], a 100% fT ≥ 10 × MIC was chosen as threshold for a dose reduction, but it is also true that supratherapeutic limits for meropenem concentrations are not well established.

A high proportion of patients presenting overexposure showed ARF and a reduced baseline eGFR at the start of the meropenem therapy, which could have caused a potential drug accumulation butthey were already receiving a lower dose adjusted to their renal function. In spite of this, a baseline eGFR lower than 90 mL/min was the only factor associated with a higher risk for achieving a supratherapeutic PK/PD target.

It has to be considered that renal function was evaluated by estimating the glomerular filtration rate (eGFR) based on Chronic Kidney Disease Epidemiology Collaboration (CKD-EPI) equation [19], which can result in overestimations in LwBW patients with low muscle bulk. However, these equations are frequently used in daily practice in the ICU [30]. Moreover, although dose adjustment was done based on renal function, a recent study showed that overexposure seems to be more frequent in patients receiving meropenem and impaired renal function [31].

One important consideration is that the high frequency of patients with excessive concentrations from a PK/PD point of view (overexposure) was not correlated with the presence of adverse events. This fact could be explained by the low frequency of patients achieving potentially toxic meropenem.

Two patients experienced DILI, but only one of them had high meropenem concentrations (80 mg/L) and previous experiences have not associated drug concentrations with liver toxicity [25]. One important limitation if that neurological toxicity could not be assessed in our sedated and mechanically ventilated patients. It is important to highlight that although β-lactams have a wide therapeutic range, precautions must be taken when high concentrations are reached because high plasma exposure has been associated with neuro- and nephrotoxicity [25].

Our study has several limitations. This is a single center, retrospective study with a limited sample size. Secondly, the potential deleterious effects of meropenem overexposure could not be adequately assessed in our population, which might have underestimated neurological toxicity. The observational nature of the study allowed to obtain only one sample timing by patient and complete concentration-time profiles and PK parameters could not be determined. Finally, although a 100% *f*T >10 × MIC has been used as dose adjustment threshold in previous studies, a supratherapeutic exposure of meropenem has to be better established.

## 5. Conclusions

Low body weight, critically ill patients treated with meropenem at standard doses by extended or continuous infusion achieved a similar plasma exposure of meropenem than normal body weight patients. No differences in the proportion of patients with a supratherapeutic PK/PD target, which was high, were observed between both groups. A baseline estimated glomerular filtration rate (eGFR) < 90 mL/min was the only factor independently associated with a supratherapeutic PK/PD target.

Our results suggest that low body weight critically ill patients treated with meropenem may not need dose adjustment based on body weight. These results should be confirmed in a future larger study assessing the PK and toxicity risk of meropenem in this population.

## Figures and Tables

**Table 1:** Demographic and clinical variables of cases and controls.

Variable	LwBW Group (Cases, *n* = 18)	NBW Group (Controls, *n* = 18)
Age (years)	61.5 (22.8)	56.5 (11.8)
Number of females, *n* (%)	8 (44)	8 (44)
APACHE-II score	15.5 (10)	19 (17)
Sepsis, *n* (%)	7 (38.9%)	2 (11.1)
Septic shock, *n* (%)	4 (22.2%)	9 (50.0)
Acute renal failure ^a^, *n* (%)	7 (38.9%)	6 (33.3)
Initial eGFR ^b^, mL/min/1.73 m^2^	92 (77)	76 (72)
Initial eGFR < 25 mL/min/1.73 m^2^, *n* (%)	1 (5.6)	1 (5.6)
Initial eGFR 25–50 mL/min/1.73 m^2^, *n* (%)	4 (22.2)	4 (22.2)
Initial eGFR 51–130 mL/min/1.73 m^2^, *n* (%)	11 (61.1)	12 (66.7)
Initial eGFR > 130 mL/min/1.73 m^2^, *n* (%)	2 (11.1)	1 (5.6)
BW ^c^, kg	45 (9.2)	84 (37)
IBW ^d^, kg	61.5 (11.8)	60.5 (14.9)
BMI ^e^, kg/m^2^	17.3 (1.6)	29.8 (17.2)
Malnutrition, *n* (%)	16 (88.9)	8 (44.4)
Total serum proteins, g/dL	4.8 (1.7)	4.9 (1.3)
Serum albumin, g/dL	2.3 (1.4)	2.5 (1.4)
Daily dose/BW of meropenem, mg/kg/day	87.5 (63.3)	62.8 (49.7)

Quantitative data are expressed as median [interquartile range]. LwBW: Low Body Weight; NBW: Normal Body Weight. ^a^ Based on the Acute Kidney Injury Network (AKIN) criteria [27]; ^b^ Initial estimated glomerular filtration rate (eGFR) Estimated by the Chronic Kidney Disease Epidemiology Collaboration (CKD-EPI) equation [19]; ^c^ BW, actual body weight; ^d^ IBW, ideal body weight by the Devine BJ formula [28]; ^e^ BMI, body mass index.

**Table 2:** PK/PD and toxicity data of the two groups.

Variable	LwBW Group (Cases, *n* = 18)	NBW Group (Controls, *n* = 18)	*p* Value
C_min,ss_, mg/L	19.9 (22.2)	22.4 (25.8)	>0.999
Optimal PK/PD			
100% *f*T_≥1–10×MIC_, *n* (%) *	6 (33.3)	3 (16.7)	0.443
Subtherapeutic levels			
100%*f*T_<MIC_, *n* (%) *	1 (5.6)	1 (5.6)	>0.999
Supratherapeutic PK/PD			
100% *f*T_>10×MIC_, *n* (%) *	11 (61.1)	14 (77.8)	0.278
Toxicity thresholds			
C_min,ss_ > 44.5 mg/L, *n* (%) **	3 (16.7)	1 (5.6)	0.603
C_min,ss_ > 64.2 mg/L, *n* (%) ***	2 (11.1)	0 (0)	0.486
DILI, *n* (%)	2 (11.1)	0 (0)	0.486

PK/PD, pharmacokinetic/pharmacodynamics; Cmin,ss: Trough or minimum concentration at steady state (expressed as median [IQR]); MIC, minimum inhibitory concentration; DILI, Drug-induced liver injury. * Considering the real MIC of the isolated pathogen or MIC value of 2 mg/L when MIC not available (EUCAST susceptibility cut-off point [26]). ** Nephrotoxicity threshold [25]. *** Neurotoxicity threshold [25].

## Data Availability

Relevant data for this study are presented in the tables. Any further data requests are available upon request from the corresponding author.

## References

[1] Nicolau D.P. (2008). Pharmacokinetic and Pharmacodynamic Properties of Meropenem. Clin. Infect. Dis..

[2] Gonçalves-Pereira J., Póvoa P. (2011). Antibiotics in critically ill patients: A systematic review of the pharmacokinetics of β-lactams. Crit. Care.

[3] Roberts J.A., Lipman J. (2009). Pharmacokinetic issues for antibiotics in the critically ill patient. Crit. Care Med..

[4] Craig W.A. (1998). Pharmacokinetic/pharmacodynamic parameters: Rationale for antibacterial dosing of mice and men. Clin. Infect. Dis..

[5] Roberts J.A., Abdul-Aziz M.H., Lipman J., Mouton J.W., Vinks A.A., Felton T.W., Hope W.W., Farkas A., Neely M.N., Schentag J.J. (2014). International Society of Anti-Infective Pharmacology and the Pharmacokinetics and Pharmacodynamics Study Group of the European Society of Clinical Microbiology and Infectious Diseases. Individualised antibiotic dosing for patients who are critically ill: Challenges and potential solutions. Lancet Infect. Dis..

[6] Roberts J.A., Ulldemolins M., Roberts M.S., McWhinney B., Ungerer J., Paterson D.L., Lipman J. (2010). Therapeutic drug monitoring of beta-lactams in critically ill patients: Proof of concept. Int. J. Antimicrob. Agents.

[7] Delattre I.K., Taccone F.S., Jacobs F., Hites M., Dugernier T., Spapen H., Laterre P.F., Wallemacq P.E., Van Bambeke F., Tulkens P.M. (2017). Optimizing β-lactams treatment in critically-ill patients using pharmacokinetics/pharmacodynamics targets: Are first conventional doses effective?. Expert Rev. Anti Infect. Ther..

[8] Abdul-Aziz M.H., Alffenaar J.C., Bassetti M., Bracht H., Dimopoulos G., Marriott D., Neely M.N., Paiva J.A., Pea F., Sjovall F. (2020). Antimicrobial therapeutic drug monitoring in critically ill adult patients: A Position Paper. Intensive Care Med..

[9] Veiga R.P., Paiva J.A. (2018). Pharmacokinetics-pharmacodynamics issues relevant for the clinical use of beta-lactam antibiotics in critically ill patients. Crit. Care.

[10] Alobaid A.S., Hites M., Lipman J., Taccone F.S., Roberts J.A. (2016). Effect of obesity on the pharmacokinetics of antimicrobials in critically ill patients: A structured review. Int. J. Antimicrob. Agents.

[11] Eisert A., Lanckohr C., Frey J., Frey O., Wicha S.G., Horn D., Ellger B., Schuerholz T., Marx G., Simon T.P. (2021). Comparison of two empirical prolonged infusion dosing regimens for meropenem in patients with septic shock: A two-center pilot study. Int. J. Antimicrob. Agents.

[12] Alobaid A.S., Wallis S.C., Jarrett P., Starr T., Stuart J., Lassig-Smith M., Ordóñez Mejia J.L., Roberts M.S., Lipman J., Roberts J.A. (2016). Effect of obesity on the population pharmacokinetics of meropenem in critically ill patients. Antimicrob. Agents Chemother..

[13] Simon P., Petroff D., Busse D., Heyne J., Girrbach F., Dietrich A., Kratzer A., Zeitlinger M., Kloft C., Kees F. (2020). Meropenem plasma and interstitial soft tissue concentrations in obese and nonobese patients—A controlled clinical trial. Antibiotics.

[14] Mahmood I. (2017). Prediction of Clearance, Volume of distribution, and Half-life of Drugs in Extremely Low to Low Birth Weight Neonates: An Allometric Approach. Eur. J. Drug Metab. Pharmacokinet..

[15] Wang Z.M., Chen X.Y., Bi J., Wang M.Y., Xu B.P., Tang B.H., Li C., Zhao W., Shen A.D. (2020). Reappraisal of the Optimal Dose of Meropenem in Critically Ill Infants and Children: A Developmental Pharmacokinetic-Pharmacodynamic Analysis. Antimicrob. Agents Chemother..

[16] Drug-Dosing-in-Extreme-of-Body-Weight. https://www.scottishintensivecare.org.uk/uploads/2014-07-24-19-55-33-Drugdosingatextremesofbod-45662.pdf.

[17] Agarwal E., Miller M., Yaxley A., Isenring E. (2013). Malnutrition in the elderly: A narrative review. Maturitas.

[18] Deurenberg P., Yap M., van Staveren W.A. (1998). Body mass index and percent body fat: A meta analysis among different ethnic groups. Int. J. Obes. Relat. Metab. Disord..

[19] Levey A.S., Stevens L.A., Schmid C.H., Zhang Y.L., Castro A.F., Feldman H.I., Kusek J.W., Eggers P., Van Lente F., Greene T. (2009). A new equation to estimate glomerular filtration rate. Ann. Intern. Med..

[20] Giangiuliani G., Mancini A., Gui D. (1989). Validation of a severity of illness score (APACHE II) in a surgical intensive care unit. Intensive Care Med..

[21] Pugh R.N., Murray-Lyon I.M., Dawson J.L., Pietroni M.C., Williams R. (1973). Transection of the oesophagus for bleeding oesophageal varices. Br. J. Surg..

[22] Thalhammer F., Hörl W.H. (2000). Pharmacokinetics of meropenem in patients with renal failure and patients receiving renal replacement therapy. Clin. Pharmacokinet..

[23] Benítez-Cano A., Luque S., Sorlí L., Carazo J., Ramos I., Campillo N., Curull V., Sánchez-Font A., Vilaplana C., Horcajada J.P. (2020). Intrapulmonary concentrations of meropenem administered by continuous infusion in critically ill patients with nosocomial pneumonia: A randomized pharmacokinetic trial. Crit. Care.

[24] Abdulla A., Ewoldt T.M.J., Hunfeld N.G.M., Muller A.E., Rietdijk W.J.R., Polinder S., van Gelder T., Endeman H., Koch B.C.P. (2020). The effect of therapeutic drug monitoring of beta-lactam and fluoroquinolones on clinical outcome in critically ill patients: The DOLPHIN trial protocol of a multi-centre randomised controlled trial. BMC Infect. Dis..

[25] Imani S., Buscher H., Marriott D., Gentili S., Sandaradura I. (2017). Too much of a good thing: A retrospective study of β-lactam concentration-toxicity relationships. J. Antimicrob. Chemother..

[26] Clinical Breakpoints-Bacteria. n.d. http://www.eucast.org/clinical_breakpoints/.

[27] Mehta R.L., Kellum J.A., Shah S.V., Molitoris B.A., Ronco C., Warnock D.G., Levin A. (2007). Acute kidney injury network: Report of an initiative to improve outcomes in acute kidney injury. Crit. Care.

[28] Pai M.P., Paloucek F.P. (2000). The origin of the “ideal” body weight equations. Ann. Pharmacother..

[29] Bergen P.J., Bulitta J.B., Kirkpatrick C.M.J., Rogers K.E., McGregor M.J., Wallis S.C., Paterson D.L., Nation R.L., Lipman J., Roberts J.A. (2017). Substantial Impact of Altered Pharmacokinetics in Critically Ill Patients on the Antibacterial Effects of Meropenem Evaluated via the Dynamic Hollow-Fiber Infection Model. Antimicrob. Agents Chemother..

[30] Baptista J.P., Neves M., Rodrigues L., Teixeira L., Pinho J., Pimentel J. (2014). Accuracy of the estimation of glomerular filtration rate within a population of critically ill patients. J. Nephrol..

[31] De Vroom S.L., van Daalen F.V., Zieck S.E., Mathôt R.A.A., van Hest R.M., Geerlings S.E. (2021). Does dose reduction of renally cleared antibiotics in patients with impaired renal function lead to adequate drug exposure? A systematic review. Clin. Microbiol. Infect..

